# Increased maximum gradient amplitude improves robustness of spin-echo cardiac diffusion-weighted MRI

**DOI:** 10.1186/1532-429X-17-S1-P388

**Published:** 2015-02-03

**Authors:** Eric Aliotta, Stanislas Rapacchi, Peng Hu, Daniel B Ennis

**Affiliations:** 1 Radiology, UCLA, Los Angeles, CA, USA; 2 Biomedical Physics IDP, UCLA, Los Angeles, CA, USA

## Background

Cardiac motion presents a major challenge in diffusion weighted MRI (DWI), often leading to large signal dropouts that necessitate repeated measurements (Pai, V.M., *MRM* 2011). While cardiac DWI is generally ECG gated to apply diffusion weighting during peak-systole or end-diastole, these intervals can be short and difficult to pinpoint, resulting in poor sequence reproducibility.

Recent improvements in gradient hardware provide larger maximum gradients than current systems (G_max_=80mT/m), which can substantially reduce the temporal footprint of diffusion preparation and make cardiac DWI more robust to bulk motion.

## Methods

A left ventricular (LV) motion model simulated motion of the healthy heart with 30-70ms quiescent intervals (t_Q_). Monopolar encoded SE-DWI (b=500 s/mm^2^, 3 directions) was simulated using: G_max_=40 and 80mT/m with diffusion gradients centered at mid-quiescence and with a range temporal offsets (ΔT=±20ms). Complex Gaussian noise was added such that SNR=50 for b=0 images. Bulk motion induced error was measured by the bias in apparent diffusion coefficient (ADC) recovery from the programmed value (ADC=1x10^-3^ mm^2^/s). Sequences that recovered ADC with bias<10% for ΔT=±10ms were deemed robust to motion.

Three healthy volunteers were scanned in a 3.0 T Siemens Prisma (Gmax=80mT/m) scanner using breath hold cardiac DWI (12 directions, monopolar encoding, single-shot SE EPI readout). Five repetitions of each sequence were acquired: 1) G40-b300: G_max_=40mT/m, b=300 mm^2^/s, TE=44ms; 2) G40-b100: G_max_=40mT/m, b=100mm^2^/s, TE=36ms; and 3) G80-b300: G_max_=80mT/m, b=300 mm^2^/s, TE=36ms. Imaging was timed to the diastolic quiescent interval, which was determined visually from CINE images.

Two observers evaluated the quality of all images as: "acceptable"-no significant signal dropouts in myocardium or "unacceptable"-significant signal dropouts, and directly compared G40-b300 to G80-b300 (fixed b-value).

## Results

**Figure 1:**
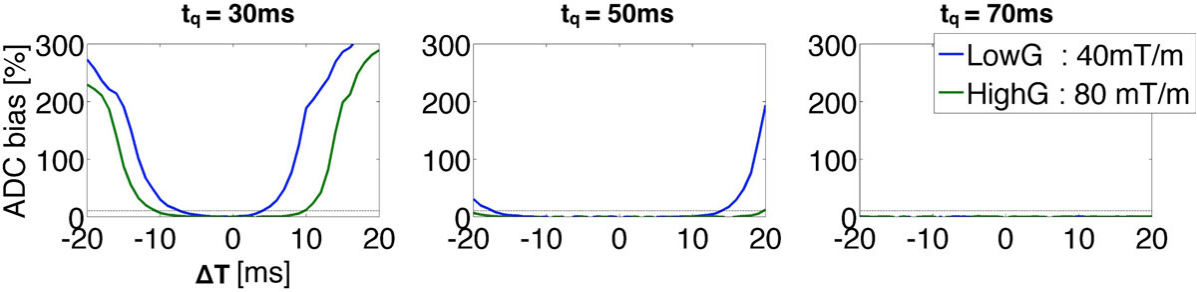
Percent ADC Bias vs. △T (temporal offsets) for different tQ (quiescent interval) for b-value=300 s/mm^2^. G80-b300 can provide acceptably small ADC bias for t_q_≥30ms, but G40-b300 requires t_q_≥50ms.

The simulated ADC bias (Fig. 1) shows that G80-b300 can provide acceptably small ADC bias for t_q_≥30ms, but G40-b300 requires t_q_≥50ms.

**Figure 2:**
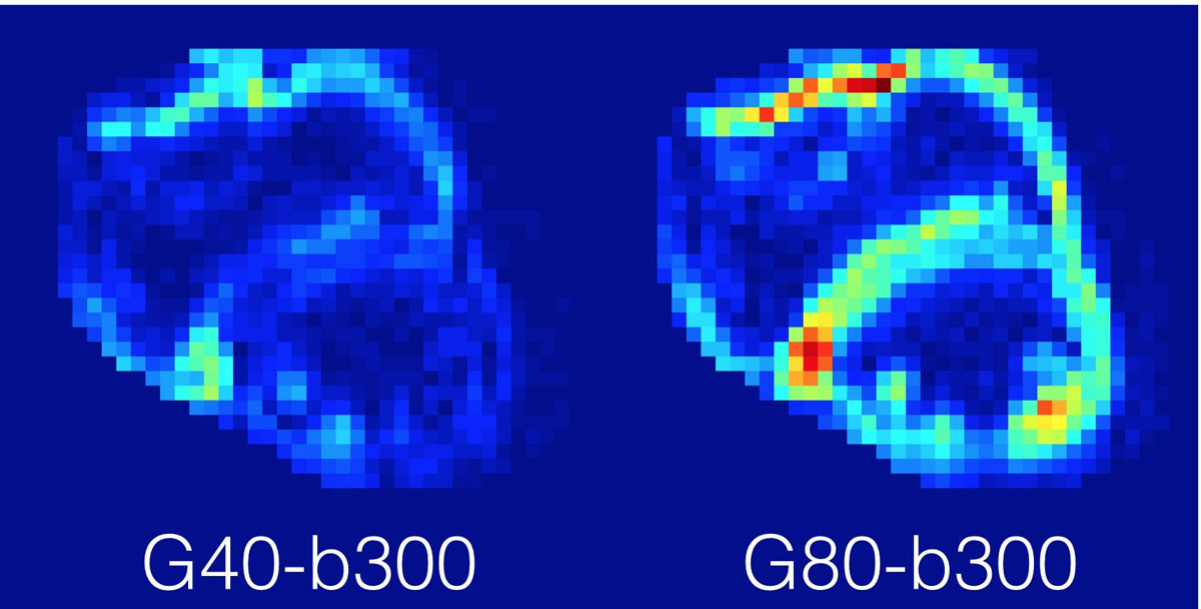
Typical DWI from G40-b300 (left) and G80-b300 (right). Image quality was generally better for G80-b300.

Image quality was better in G80-b300 than G40-b300 in 86% of images (example pair shown in Figure 2). 55% of G80-b300 images were acceptable, whereas 30% of G40-b300 and 70% of G40-b100 were.

## Conclusions

Simulations show that G80 recovered ADC more accurately than G40 for all t_Q_ and ΔT and was robust to motion for t_Q_≥30ms. This is likely due to the shorter diffusion preparation (G40 t_prep_=39ms, G80 t_prep_=28ms) and indicates that G80 will perform more consistently for short t_Q_ (fast heart rates, systolic imaging) or changes in heart rhythm.

With fixed b-value=300mm^2^/s in vivo, G80 had consistently better image quality than G40. In agreement with simulation, this indicates that G80 improves the robustness of cardiac DWI for the same b-value. With fixed TE=36ms, G40-b100 was acceptable more frequently than G80-b300, but with insufficient diffusion weighting. Increased G_max_ can thus improve diffusion sensitivity with less loss of robustness.

## Funding

This research was supported by Siemens Medical Solutions and the Department of Radiological Sciences at UCLA.

